# Impact of timing for intra-aortic balloon pump insertion on patients undergoing mitral valve surgery

**DOI:** 10.3389/fcvm.2026.1717294

**Published:** 2026-03-09

**Authors:** Liuyi Du, Xiaofeng Xu, Huanlei Huang, Manxia Xing, Miaoyun Chen, Xuejun Xie

**Affiliations:** Department of Heart Valve & Coronary Artery Surgery, Institute of Cardiovascular Disease, Guangdong Provincial People’s Hospital (Guangdong Academy of Medical Sciences), Southern Medical University, Guangzhou, China

**Keywords:** intra-aortic balloon pump, mitral valve, mortality, surgery, timing

## Abstract

**Background:**

This study aimed to investigate the timing of intra-aortic balloon pump (IABP) insertion in patients undergoing mitral valve surgery.

**Methods:**

A total of 136 patients who had undergone mitral valve surgery and IABP insertion under cardiopulmonary bypass at Guangdong Provincial People's Hospital between June 2017 and June 2021 were enrolled in this retrospective analysis. The patients were divided into early and late IABP insertion groups based on the timing of the insertion. The primary endpoint of the observation was the survival rate of patients in both groups, while the secondary endpoints included the incidence of complications and the length of hospital stay.

**Results:**

Among the 136 patients enrolled, 55 were in the early insertion group and 81 in the late insertion group. There was no statistically significant difference in in-hospital mortality between the two groups (*p* = 0.715). However, statistically significant differences were observed in the incidence of postoperative complications, specifically infection (*p* = 0.03) and re-exploration for bleeding (*p* = 0.02). Although pairwise comparison did not show any statistically significant difference in the length of hospital stay, the duration of intensive care unit stay was significantly shorter among patients in the early IABP insertion group (*p* = 0.01).

**Conclusions:**

The timing of IABP insertion did not exhibit significant impacts on patients undergoing mitral valve surgery.

## 1 Introduction

Low cardiac output syndrome (LOS) following cardiac surgery is associated with high mortality rates. Despite receiving substantial vasopressor support, some patients fail to achieve the expected hemodynamic response, necessitating the use of mechanical circulatory support devices. Currently, extracorporeal membrane oxygenation (ECMO), left ventricular assist devices, percutaneous mechanical circulatory support, biventricular assist devices, and intra-aortic balloon pumps (IABP) are among the most common mechanical circulatory support devices (1). Among these, IABP has emerged as the most frequently employed mechanical support device for postoperative LOS due to its safety, ease of use, and convenient insertion (2). In patients with preoperative low left ventricular ejection fraction (LVEF) and LOS undergoing cardiac surgery, perioperative IABP insertion has been shown to improve cardiac output by 10%–30% (1). This improvement is achieved by deflating the balloon during cardiac systole and inflating it during diastole, thereby increasing the pressure gradient from the aorta to the coronary circulation and enhancing myocardial perfusion (3). Over the past decade, IABP utilization has undergone changes due to the results of a large-scale randomized trial and observational data, which have sparked a growing interest in investigating the role of IABP (4). However, these investigations have primarily focused on patients undergoing coronary artery bypass grafting and percutaneous coronary intervention, with relatively limited studies focusing on patients undergoing mitral valve surgery. Moreover, in addition to assessing IABP efficacy, there is a need for further exploration of the optimal timing for IABP insertion. Therefore, this study aimed to investigate the impact of IABP insertion timing on patients undergoing mitral valve surgery.

## 2 Materials and methods

### 2.1 General information

This retrospective study analyzed patients who had undergone mitral valve surgery with perioperative IABP insertion at the authors’ institution between June 2017 and June 2021. The patients were divided into early (*n* = 55; pre- and intraoperative) and late (*n* = 81; postoperative) IABP insertion groups based on the timing of the insertion. The research protocol was approved by the Ethics Committee of Guangdong Provincial People's Hospital (approval no: KY2023-256). The study included patients who had undergone isolated mitral valve surgery with IABP insertion during cardiopulmonary bypass, including mechanical or biological mitral valve replacement and mitral valve repair, and were aged 18–70 years. None of the patients underwent IABP implantation before surgery. The study excluded patients who had undergone simultaneous procedures on other heart valves or blood vessels during mitral valve surgery, had a history of cardiopulmonary resuscitation, or had significant comorbidities like tumors.

### 2.2 IABP insertion criteria

Preoperative and intraoperative IABP insertion were indicated for patients with poor preoperative cardiac function or difficulty weaning from cardiopulmonary bypass after surgery. Alternatively, postoperative IABP insertion was indicated for patients who developed LOS and low LVEF upon returning to the intensive care unit (ICU) after surgery, despite receiving significant doses of vasopressor agents with limited therapeutic effect.

### 2.3 IABP insertion procedure

For IABP insertion preparation, a balloon catheter was first selected based on the patient's height. The insertion depth was typically set to reach the sternal angle. The balloon catheter was then flushed with heparinized saline through its central lumen, and low-molecular-weight heparin was administered subcutaneously. Under local anesthesia, the catheter was percutaneously inserted through the right femoral artery. The balloon catheter was slowly inserted along the guidewire and then connected to the IABP. With the trigger mode set to synchronize with the R-wave on the electrocardiogram, the patient's blood pressure and heart rate were closely monitored, and the inflation-to-deflation ratio was promptly adjusted as needed.

### 2.4 Clinical data collection

Relevant clinical data, including preoperative and postoperative (on days 1 and 3) levels of creatinine and aspartate aminotransferase, were collected. Additionally, clinical observation indicators, such as patients’ age, weight, cardiopulmonary bypass time, aortic cross-clamp time, and length of ICU stay, were documented. Preoperative conditions and postoperative complications of the patients were also recorded.

### 2.5 Statistical method

Data were analyzed using SPSS 26.0 and R (version 4.2.1). The variables that followed a normal distribution were presented as the mean ± standard deviation, and the intergroup comparison was performed using the independent samples *t*-test. The non-normally distributed variables were presented as the median (interquartile range), and the intergroup comparison was conducted using the Mann–Whitney *U* test. The count data was presented as relative numbers, and the intergroup comparison was conducted using the chi-squared test or Fisher's exact test. Logistic regression analysis was used to identify significant variables related to mortality, and *p* < 0.05 was considered statistically significant. The independent variables that were statistically significant in the univariate Cox regression analysis (*P* < 0.05) were incorporated into the multivariate Cox regression analysis. Variables that remained statistically significant in the multivariate model (*P* < 0.05) were then used to construct and plot the nomogram. The presented nomogram was derived solely from the coefficients of the multivariable logistic regression model.

## 3 Results

### 3.1 General information

The patients were divided into early and late IABP insertion groups based on the timing of insertion. The baseline characteristics of the patients are presented in Table 1. Out of the 136 patients, 51 were included in the early insertion group, and 81 were in the late insertion group. There was a statistically significant difference in gender distribution between the two groups, with a higher proportion of males in the late IABP insertion group (*p* = 0.01). However, no statistically significant differences were observed in other baseline characteristics between the two groups. The median weight was 48 kg in the early IABP insertion group and 40 kg in the late IABP insertion group. The median age of the early and late IABP insertion groups was 61 and 62 years, respectively. The incidence of comorbidities, such as hypertension, diabetes, and chronic kidney disease, in the two groups was not statistically significant.

**Table 1:** Baseline characteristics of the two groups of patients.

Variable	Early IABP Insertion (*n* = 55)	Late IABP Insertion (*n* = 81)	*p*
Gender			0.01
Female	31 (56.36)	26 (32.09)	
Male	24 (43.64)	55 (67.90)	
Alcohol consumption	6 (10.91)	10 (12.35)	0.80
Smoking	4 (7.27)	14 (17.28)	0.09
Weight (kg)	48 (0–55)	40 (0–55)	0.23
Age (years)	61 (55–66)	62 (54–67)	0.79
Creatinine (μmol/L)	89 (73, 116)	81 (67, 101)	0.073
AST (U/L)	19 (15–29)	18 (14–28)	0.32
Comorbidities			
Chronic renal disease	1 (1.82)	1 (1.24)	0.78
Chronic obstructive pulmonary disease	3 (5.46)	8 (9.88)	0.35
Diabetes mellitus	7 (12.73)	8 (10.00)	0.62
Hypertension	17 (30.91)	18 (22.22)	0.26
Stroke	1 (1.82)	1 (1.24)	0.78
LV EF	56.47 ± 10.86	56.83 ± 9.93	0.845
blood pressure	111.15 ± 19.24	110.64 ± 21.83	0.890
heart rate	89.73 ± 12.96	94.65 ± 16.74	0.074
CO	3.65 (3.20, 4.60)	3.20 (2.60, 3.60)	0.251
Lactate	6.50 (3.02, 11.00)	5.70 (3.40, 8.50)	0.529
Vis	15.00 (8.50, 20.75)	11.00 (8.00, 20.00)	0.694

Data are presented as *n* (%), median (interquartile range), or mean ± standard deviation.

AST, aspartate aminotransferase; IABP, intra-aortic balloon pump. CO, Cardiac Output. Vis, Vasoactive-Inotropic Score.

### 3.2 Intraoperative status and postoperative complications

The intraoperative and postoperative details of the patients are presented in Table 2. Statistically significant differences were observed in the techniques used for mitral valve treatment between the two groups, including mitral valve repair, mechanical mitral valve replacement, and biological mitral valve replacement. Specifically, the proportion of patients undergoing mitral valve repair was higher in the early IABP insertion group, whereas the late IABP group had a higher proportion of patients undergoing mechanical and biological mitral valve replacement (*p* = 0.002). There were no statistically significant differences in the cardiopulmonary bypass time and aortic cross-clamp time between the two groups (*p* = 0.15). However, the length of ICU stay for patients in the early IABP insertion group was substantially lower than that of patients in the late IABP insertion group (*p* = 0.01). Nevertheless, the overall length of hospital stay was not statistically significant between the two groups (*p* = 0.55). Regarding postoperative complications, no statistically significant differences were observed between the two groups in terms of stroke, tracheal intubation, or wound healing complications. Although the incidence of dialysis showed notable differences between the two groups, this difference was not statistically significant (*p* = 0.06). The proportion of patients requiring dialysis was 21.82% in the early IABP insertion group and 37.04% in the late IABP insertion group. Additionally, the postoperative infection rates between the two groups were statistically significant (*p* = 0.03).

**Table 2:** Intraoperative and postoperative conditions of the two groups of patients.

Variable	Early IABP Insertion (*n* = 55)	Late IABP Insertion (*n* = 81)	*p*
Valve treatment technique			0.002
Mitral valve repair	27 (33.33)	4 (4.94)	
Mechanical mitral valve replacement	24 (43.64)	50 (61.73)	
Biological mitral valve replacement	17 (30.91)	27 (33.33)	
CPB (minutes)	190.25 ± 74.18	172.79 ± 57.80	0.15
ACC (minutes)	100.84 ± 37.71	100.37 ± 37.50	0.95
ICU stay (days)	12 (8–15)	15 (11–21)	0.01
Hospital stay (days)	33 (25–42)	31 (25–49)	0.55
Mortality	13 (23.636)	17 (20.988)	0.715
Stroke	1 (1.82)	1 (1.82)	0.78
Dialysis	12 (21.82)	30 (37.04)	0.06
Tracheal intubation	3 (5.46)	8 (9.88)	0.35
Infection	12 (22.22)	33 (40.74)	0.03
Poor wound healing	5 (9.09)	15 (18.52)	0.13
Re-exploration for bleeding	54 (98.182)	70 (86.420)	0.02
Extracorporeal membrane oxygenation	8 (14.545)	8 (9.877)	0.41
AST on postoperative day 1 (U/L)	29 (19.5- 44.5)	32 (18.75–73.5)	0.55
AST on postoperative day 3 (U/L)	29 (20–57)	36 (18.75–145.25)	0.68
Creatinine on postoperative day 1 (μmol/L)	120 (99–148)	108 (85.75–148.25)	0.131
Creatinine on postoperative day 3 (μmol/L)	135 (106.5–187.5)	132.5 (96.25, 195.25)	0.826

Data are presented as *n* (%), median (interquartile range), or mean ± standard deviation.

AST, aspartate aminotransferase; IABP, intra-aortic balloon pump; ICU, intensive care unit.

### 3.3 Uni- and multivariate analyses of mortality

**Figure 1:**
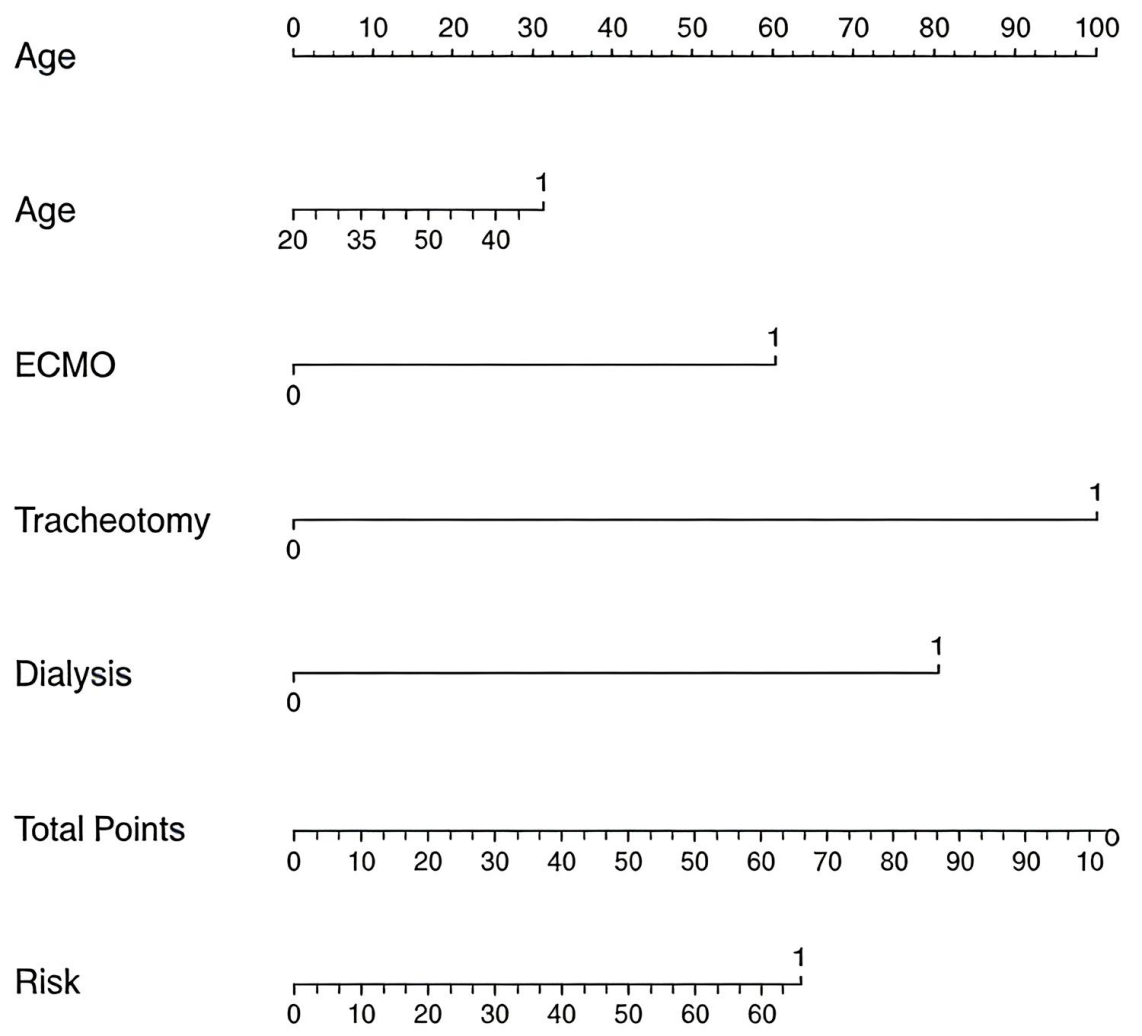
Multivariate nomogram of the influencing factors on mortality.

Table 3 presents the analysis of factors influencing the mortality rate among patients with IABP insertion. For each categorical variable, the baseline was set as females, early IABP insertion, absence of hypertension, absence of diabetes, non-smokers, non-drinkers, no history of stroke or chronic obstructive pulmonary disease, and patients who did not undergo postoperative dialysis, ECMO assistance, or tracheostomy. The univariate logistic regression analysis revealed that age, postoperative dialysis, ECMO assistance, and postoperative tracheostomy were significantly associated with mortality (*p* < 0.05). Further multivariate logistic regression analysis of these four factors revealed that postoperative dialysis, ECMO assistance, and postoperative tracheostomy were significant predictors of mortality (*p* < 0.05). Therefore, postoperative dialysis [odds ratio [OR] = 11.22; 95% confidence interval [CI] = 3.79–38.62], ECMO assistance (OR = 7.43; 95% CI = 1.90–33.13), and postoperative tracheostomy (OR = 20.45; 95% CI = 4.01–132.50) were identified as risk factors for mortality. Figure 1 shows the nomogram depicting the multifactorial influences on mortality.

**Table 3:** Uni- and multivariate analyses of mortality.

Variable	Univariate analysis		Multivariate analysis	
	OR (95% CI)	*p*	OR (95% CI)	*p*
Male	0.93 (0.41–2.14)	0.86		
Body weight	1.01 (1.00–1.03)	0.21		
Age	1.05 (1.01–1.11)	0.04	1.02 (0.96–1.08)	0.57
Hypertension	2.13 (0.84–5.19)	0.10		
Diabetes	1.32 (0.34–4.21)	0.66		
Smoking	0.40 (0.60–1.53)	0.24		
Alcohol consumption	2.40 (0.75–7.14)	0.12		
History of stroke	2.24 (0.44–9.74)	0.29		
History of COPD	0.77 (0.11–3.21)	0.75		
Preoperative creatinine	1.00 (0.99–1.00)	0.42		
Preoperative AST	1.00 (1.00–1.01)	0.31		
Postoperative dialysis	11.83 (4.77–32.05)	0.00	11.22 (3.79–38.62)	0.00
ECMO	11.70 (3.81–40.82)	0.00	7.43 (1.90–33.13)	0.00
ACC	1.00 (0.99–1.01)	0.94		
CPB	1.00 (1.00–1.01)	0.20		
IABP timing	0.86 (0.38–1.98)	0.72		
Postoperative tracheostomy	12.49 (3.33–60.56)	0.00	20.45 (4.01–132.50)	0.00

Data are presented as *n* (%), median (interquartile range), or mean ± standard deviation.

AST, aspartate aminotransferase; COPD, chronic obstructive pulmonary disease; ECMO, extracorporeal membrane oxygenation; IABP, intra-aortic balloon pump.

## 4 Discussion

An IABP is a mechanical circulatory support device that enhances coronary blood flow and improves systemic hemodynamics. It is widely used in patients undergoing cardiac surgery who develop LOS and have a reduced LVEF. The utilization of IABP is more extensive in Asian countries, making it the most common mechanical circulatory device used during the perioperative period of cardiac surgeries. However, the effectiveness and optimal timing of IABP insertion remain controversial and are a frequently discussed topic (5).

The application of IABP during the perioperative period of valve surgery has not been extensively studied. Therefore, our study aimed to investigate the timing of IABP insertion in patients with mitral valve disorders during the perioperative period. We compared the clinical effects of early and late IABP insertions and found that the insertion timing did not impact the in-hospital survival or length of hospital stay for patients undergoing mitral valve surgery. Nevertheless, the incidence of postoperative complications was affected by the timing, with a higher proportion of patients requiring postoperative hemodialysis in the late IABP insertion group. However, the pairwise difference was not statistically significant, warranting further validation with an increased sample size. Compared to late IABP insertion, early IABP insertion offers a certain degree of renal protection for patients undergoing mitral valve surgery. Additionally, patients who underwent early IABP insertion demonstrated enhanced recovery during their ICU stay, resulting in a shorter ICU stay.

The use of IABP remains controversial in current studies, and guidelines discourage its use in cases of myocardial infarction based on a single randomized trial published in 2012. The trial investigated the effect of IABP on patients using a series of primary and secondary endpoints, including all-cause mortality, renal function, arterial lactate levels, and vasopressor use (6, 7). Subsequent research has further investigated the role of IABP. A meta-analysis summarizing 23 articles comparing IABP insertion with traditional treatment methods found that four studies reported a decrease in mortality with IABP insertion and seven studies showed a significant increase in mortality with IABP insertion, while other studies did not report a significant difference (8). In a randomized controlled study involving patients with cardiogenic shock, IABP insertion was compared to drug therapy (mainly using levosimendan or dobutamine) based on variables like central venous oxygen saturation, 30-day mortality rate, cumulative fluid balance, and severity of respiratory distress. The results of this study indicated that IABP insertion was superior to drug support therapy (9).

The timing of IABP insertion has also been a subject of discussion. Poirier et al. conducted an analysis of 46,067 patients and concluded that preoperative IABP insertion could reduce in-hospital mortality and the length of ICU stay, although the benefits of intra- or postoperative IABP were limited (10, 11). In a review by Deppe et al. involving 9,212 patients who underwent coronary artery bypass grafting, preoperative IABP insertion offered greater benefits. Compared to late IABP insertion, preoperative IABP insertion not only significantly reduced mortality as well as the incidence of renal failure, myocardial infarction, and other complications, but also lowered the risks of hospitalization and ICU admission (12). Gul et al. conducted a retrospective analysis of 193 patients with cardiogenic shock who underwent IABP treatment, with the exclusion of patients requiring subsequent ECMO. The findings revealed that patients who underwent IABP within one hour after identifying cardiogenic shock had lower mortality rates compared to those who underwent late IABP insertion. Additionally, less vasopressor use was required by patients who underwent early IABP insertion (13).

Conversely, a study conducted in 2013 on patients undergoing percutaneous coronary intervention (PCI) after myocardial infarction found that late IABP insertion provided greater benefits to patients compared to early IABP insertion. The study observed that patients who had IABP inserted before PCI had higher creatine kinase levels, indicating a larger infarct size, suggesting that the outcomes were not as favorable as with late IABP insertion (14).

The results of our study share both similarities and differences with previous studies. Our study specifically targeted patients undergoing mitral valve procedures, acknowledging that the impact of IABP could vary depending on different cardiac conditions. We found that although early IABP insertion exhibited minimal effects on patients’ length of hospital stay and mortality, it reduced the incidence of complications. In earlier studies, hypotension was typically considered a significant marker for identifying cardiogenic shock. However, more recently, the evaluation of inadequate perfusion has caught significant attention. This is because, in some cases, perfusion deficits caused by low cardiac output may not be readily apparent due to pharmacological support and assistance from mechanical devices. In such instances, the patient's perfusion status can be assessed by evaluating renal function and lactate levels (15–17). Our study revealed a lower proportion of patients requiring blood dialysis in the early IABP insertion group, suggesting improved perfusion status. The proportion of patients experiencing infections was relatively higher in the late IABP insertion group. This can possibly be attributed to the fact that early IABP insertion often occurs in the theater, whereas late IABP insertion often takes place in the ICU with a worse sterile environment, influencing patients’ infection rates. This subsequently affects patients’ treatment in the ICU, necessitating an extended ICU stay due to renal function and infection status. However, in general, for patients undergoing mitral valve procedures, the timing of IABP insertion exhibited a relatively minor impact on survival outcomes and overall length of hospital stay.

To eliminate the influence of specific factors on survival rates, multiple logistic regression was employed to identify the independent factors associated with in-hospital mortality. The results of the analysis suggested that the timing of IABP insertion was not a significant factor for mortality, demonstrating its limited impact on patient survival.

### 4.1 Study limitations

Our study was conducted at a single center, which might have introduced some bias in the results. To better explore the impact of IABP insertion on patients, further research involving multiple centers and a larger sample size is required. Additionally, long-term follow-up was not performed in the study, making it infeasible to understand the effect of the timing of IABP insertion on the long-term prognosis of valve patients. Lastly, this study only included patients undergoing mitral valve procedures, limiting the generalizability of the findings to other cardiac surgery patients who undergo IABP insertion. The retrospective nature of the study and possibly low power for a multivariable analysis.

## Data Availability

The raw data supporting the conclusions of this article will be made available by the authors, without undue reservation.
